# Adaptation of a psycho-educational group programme to improve coping in dementia caregiving: a feasibility study with mixed-methods

**DOI:** 10.1186/s12877-024-04815-7

**Published:** 2024-02-27

**Authors:** Sandrine Pihet, M. Clément, E. Terrapon, S. Kipfer

**Affiliations:** 1 School of Health Sciences, HES-SO University of Applied Sciences and Arts Western Switzerland, Fribourg, Switzerland; 2 Haute Ecole de Santé Fribourg, Route Des Arsenaux 16a, 1700 Fribourg, Switzerland

**Keywords:** Dementia, Informal caregiver, Psycho-educational intervention, Stress, Coping strategies

## Abstract

**Background:**

As the number of people living with dementia rapidly increases worldwide, the support provided by their informal caregivers remains key to the sustainability of most healthcare systems, this voluntary contribution representing 40% of the costs of dementia worldwide. Informal caregiving in dementia, however, is linked to long periods of chronic stress with frequent and serious negative consequences on the health and quality of life of the caregiver. A psycho-educational group intervention focusing on coping with the daily stress of dementia caregiving (“Learning to feel better… to help better”), developed in French-speaking Canada and showing broad effects on quality of life, was selected with the aim of 1) adapting it to a new cultural context (French-speaking Switzerland) based on identified facilitators and barriers, using a participative approach; and 2) conducting a feasibility study to evaluate whether the adapted programme showed similar or improved feasibility and effects compared to the original Canadian programme.

**Methods:**

A mixed-methods concurrent nested design was used to evaluate the feasibility and the effects on five quantitative core outcomes. Additional qualitative data helped document in depth the acceptability and impact of the intervention.

**Results:**

We shortened the programme from 30 to 21 h in total, which resulted in increased accessibility, in terms of facilitated recruitment of participants and inclusion of a broader range of informal caregivers. There were significant reductions in subjective burden (effect size: *d* = -0.32) and psychological distress (*d* = -0.48), as well as decreases in the stress reactions of informal caregivers related to the behaviour problems of the persons with dementia (*d* = -0.57). The qualitative results emphasized the usefulness of providing informal caregivers with structured procedures for efficiently tackling everyday challenges, and of enabling learning through a variety of channels and activities.

**Conclusions:**

Substantial improvements are associated with this 21-h group intervention, organised in 7 sessions of 3 h each, focused on learning more efficient strategies to cope with the daily stress of dementia caregiving. This intervention empowered informal caregivers to master their daily challenges with more confidence, satisfaction and calm.

**Trial registration:**

ISRCTN13512408 (registration date 17.05.2021, retrospectively registered).

**Supplementary Information:**

The online version contains supplementary material available at 10.1186/s12877-024-04815-7.

## Background

Worldwide, over 50 million people were living with dementia in 2020, with over 10 million new cases each year [1]. Due to population ageing, the number of affected persons is expected to almost double every 20 years, reaching 82 million in 2030 and 152 million in 2050 [2]. In Switzerland, where the population is among the oldest on the planet, 146,500 people were living with dementia in 2020, with one new person diagnosed every 17 min [3]. For each person with dementia (PwD), between one and three relatives are involved in providing assistance, supervision and care, which increase in intensity over the course of the disease [3]. These informal caregivers of a person with dementia (ICD), often the spouse or adult children, spend on average 5.7 h a day in caregiving; female ICD contribute 70% of these hours [4]. ICD voluntary involvement is key to the sustainability of most health care systems as it covers about 40% of the costs of dementia worldwide [4], and 47% in Switzerland [5].

The sustainability of the contribution of ICDs is a core public health issue, as taking up such a role is becoming more difficult due to shifting family structures (e.g. smaller family size, family members living further apart, blended family), as well as higher employment rates for women and increasing professional pressure on all workers [6]. Although informal caregiving is often considered rewarding as it provides a sense of personal accomplishment and strengthens relationships [7], it also imposes high demands and costs, particularly for those involved in dementia care. Many ICDs experience long periods of chronic stress and a heavy subjective burden, reduced quality of life and social isolation, as well as increased physical and mental health challenges, compared to their non-caregiving counterparts or to caregivers of people without dementia [e.g. [8, 9]. ICD subjective burden and health deterioration are core predictors of early institutionalisation [10] and mistreatment [11] of their care recipient. In Switzerland as in many other high-income countries, a diversity of support options are available for PwD and their ICDs. Most of the support focuses on diagnosis, treatment and care for the PwD, as well as respite and information for the ICD [﻿[12], for more details see [13]]. Training opportunities supporting ICD self-management are less common, despite their relevance to prevent the exhaustion of ICDs and protect their quality of life [14].

Meta-analyses show that psycho-educational interventions have the broadest effects compared to other forms of support [15, 16] and that focusing on coping strategies with a psycho-educational group intervention holds the most promise for countering chronic stress in ICDs [e.g. [17]. Indeed, a recent meta-analysis of 56 multifactorial studies confirmed that coping strategies and self-efficacy were core and highly stable predictors of subjective caregiver burden [18]. Coping with the daily stress of dementia caregiving is the main focus of the psycho-educational group programme “Learning to feel better… to help better” (LFBHB) developed in Quebec, Canada. This programme was first tested in a randomized controlled trial across six centres in Quebec with 116 ICDs; It was found effective in decreasing the frequency of behaviour problems in the PwD and associated distress in the ICD, with respective effect sizes of *d* = 0.09 and *d* = 0.38 [19].

### Aims of the study

The current publication provides an overview over the different steps undertaken to develop a psycho-educational intervention for ICDs. It mainly reports how the LFBHB intervention has been adapted to the Swiss French-speaking context with a participative approach and then evaluated within a feasibility and pilot trial with a single group pre- and post-test design. The adaption phase aimed to tailor the programme to the Swiss context based on identified facilitators and barriers. The evaluation phase aimed to evaluate whether the adapted LFBHB programme showed improved feasibility and similar effects compared to the original LFBHB intervention.

## Methods

**Fig. 1:**
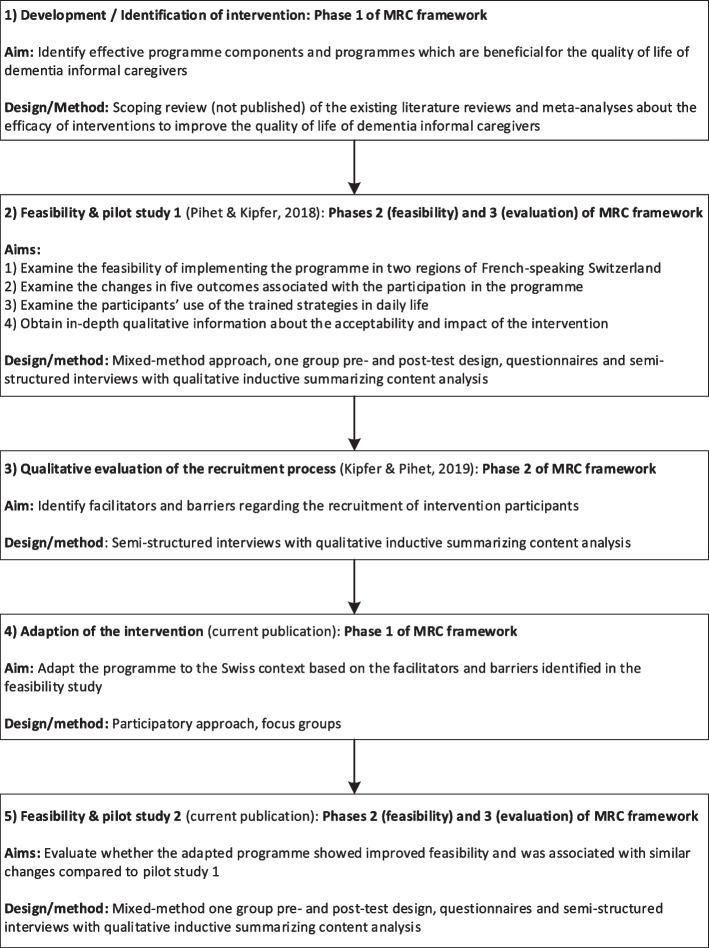
Steps to develop the complex intervention in relation to the phases of the MRC framework

In order to develop a psycho-educational intervention for ICDs we conducted several steps belonging to the first three phases of the Medical Research Council (MRC) framework for the development of complex interventions [20], namely 1) develop or identify intervention, 2) feasibility, and 3) evaluation. Figure 1 provides an overview of the different steps. This article describes in detail the rationale, methods and results of step 4 (adaption of the intervention) and step 5 (feasibility & pilot study 2). To provide background information, it also presents shortly step 1 (development of the intervention), step 2 (feasibility and pilot study 1), and step 3 (qualitative evaluation of the recruitment process).

### Step 1: Development / identification of the intervention

We conducted a scoping literature review to identify effective interventions for improving the quality of life of ICDs. The psycho-educational group programme “Learning to feel better… to help better” (LFBHB) developed in Quebec, Canada, has been identified as the sole intervention in French supported by empirical evidence [19]. This programme of 15 weekly sessions of two hours each focuses on five main themes: 1) Daily life organisation and communication with a PwD; 2) The appraisal of stressful situations, identification of what is modifiable versus non-modifiable and choice of relevant coping strategies; 3) Problem solving in modifiable situations, in particular for problem behaviours of the PwD; 4) Reframing in non-modifiable situations, in particular for thoughts and emotions related to mourning; 5) Finding and asking for support (for more detail see the methods section).

### Step 2: Feasibility and pilot study 1

In a next step, we evaluated the feasibility of the LFBHB programme in a different cultural context, French-speaking Switzerland, with a one-group pre-post design involving 18 ICDs across two regions [13]. The programme was well accepted, with high participation rates (on average, ICDs took part to 92% of the sessions) and low dropout rates (21%). Participants showed substantial and significant improvements in burden (*d* = 0.41), psychological distress (*d* = 0.54) and self-efficacy (*d* = 0.43). However, recruitment was a challenge, mostly due to the length of the programme [13].

### Step 3: Qualitative evaluation of the recruitment process

To identify barriers and facilitators to participation, we consequently performed a qualitative evaluation of the recruitment process [21, 22] at the end of our first pilot trial testing the feasibility of the original LFBHB programme [13].

#### Methods

We conducted semi-structured interviews with 14 professionals and one volunteer (11 women, 4 men; professionals were 10 nurses, 2 social workers, 1 dietician) employed in 10 different institutions, such as memory clinics, homecare agencies, day-care centres, the Alzheimer Association and the Red Cross. The sole inclusion criteria was being actively involved in the recruitment of LFBHB participants and thus having regular contact with informal caregivers. The questions focused on facilitators and barriers to participation in the context of the recruiter’s institution, of their relationship with ICDs and of ICD’s reactions to the information provided about the intervention. Interviews lasted on average 40 min (min = 21, max = 62), were audio recorded, verbatim transcribed and verified. We conducted a content analysis with inductive category based on Mayring [23].

#### Results

We identified three categories [for details, see [21, 22]: 1) compatibility with everyday life and resources, 2) finding the right moment for participation and 3) familiarity of the recruiter as a facilitator for participants’ involvement. Regarding 1) compatibility with everyday life and resources, a major obstacle to participation was the duration of the programme. Many ICDs were reluctant to commit for 15 weeks as they were living from day to day due to a highly unpredictable and demanding context. Regarding 2) finding the right moment for participation, many recruiters first came in contact with the ICD when the latter was already stressed and exhausted. At such a time, respite was a priority. Taking part in a psycho-educational programme, which requires resources to develop new competencies, was seen as additional stress. Regarding 3) familiarity of the recruiter as a facilitator for participants’ involvement, recruiters with a good knowledge of and a trusting relationship with ICDs found it easier to identify those who could benefit from the programme and provide them with adapted information, while the ICDs listened to them with confidence. Recruiters with a good knowledge of the programme were able to provide detailed and relevant information, while those with less knowledge often presented LFBHB as one support option among others.

### Step 4: Adaptation of the intervention

Based on the qualitive findings regarding the recruitment process, the programme was adapted, with a participatory approach, to facilitate recruitment.

#### Methods

Seven experts in the field of dementia caregiving composed our multidisciplinary advisory team (psychology, nursing, social work, occupational therapy). These experts were active in diverse environments (applied research, education in nursing, memory clinic, day-care centres, Alzheimer Association) in two cantons of Switzerland (Fribourg and Jura). All experts were familiar with the LFBHB programme. Based on the results of the qualitative study, this advisory team elaborated concrete propositions for improving recruitment and for shortening the programme. The propositions on recruitment were then submitted to 4 other professionals from local memory clinics (1 geriatrician, 1 neuropsychologist) and homecare agencies (2 nurses). We also presented the propositions for programme shortening to 1) 5 ICDs (5 women; 4 spouses and 1 sister) with experience in the LFBHB programme, within a focus group, and 2) 4 others experienced LFBHB course leaders from 3 regions of Switzerland and from Québec (Canada), within semi-structured individual interviews. All the discussions were audio recorded and the expressed opinions were summarized.

#### Results

All experts and ICDs were in favour of keeping the five main themes of the programme. All of them further agreed that the didactic methods should globally be maintained, namely providing information about one theme with its practical tools, then applying these tools to the stressful situations of the course participants, and in addition encouraging the latter to use these tools at home between course sessions (homework). All participants further agreed on the importance of asking experienced caregivers who had already completed the course to share their knowledge with course participants. As regularly telling their live stories during the course would put an additional burden on the experienced caregivers, all experts and all ICDs considered the use of filmed stories an excellent alternative. All experts further found that the written A4 flyer used by recruiters to provide information to possible participants should be complemented by a short film presenting the program with concrete examples from former participants. This medium should also facilitate access for people with diverse educational backgrounds.

Practically, we identified a consensus for shortening the programme and improving recruitment by three means:

1. Before the start of the programme, raising the ICDs’ awareness about stress and coping by showing them a 30-min introduction video film about stress and coping in the context of dementia caregiving. This video presents in detail the importance of stress and coping in the context of dementia caregiving, and the strategies taught in the course (e.g., problem-solving for challenging behaviours of the PwD, seeking support), illustrated by practical examples from the daily life of five former participants. Other video sequences show how participants perform practical exercises during the course session with the help of the group leader. This material provides a concrete overview of what participants can expect from the programme. A web link with free access to this film was provided to all recruiters so that they could watch it as a training before starting the recruitment, and then provide this link to ICDs interested in this intervention.
2. During the programme, a) condensing and standardising information delivery by using short didactic video films (5 videos of 8 to 15 min, one per theme) including stories of how former participants applied the programme strategies in their daily life, to facilitate transfer, and b) during the group sessions, limiting the number of exercises for all themes (1–2 instead of 2–3) as well as conducting some exercises in subgroups so that more participants can be active. These changes helped reduce the number of sessions to 7 (instead of 15) with a slightly longer session duration of 3 h (instead of 2 h), for a total of 21 h (instead of 30 h).
3. After the programme, offering additional opportunities to exercise during booster sessions, so that all participants have a sufficient opportunity to practice the programme strategies despite the reduced duration and get continuing support after the end of the intervention.

### Step 5: Feasibility and pilot study 2

Following the adaption, we conducted a second feasibility and pilot trial with a single group pre- and post-test design in two regions of French-speaking Switzerland. The aim was to evaluate if the shortened intervention showed improved feasibility while maintaining similar effects to those observed in the first feasibility study. Regarding improved feasibility, we expected 1) an efficient recruitment process (easy recruitment of 7 to 10 participants per group, few refusals linked to the length of the programme), 2) high participation and low drop-out rates during the programme, and 3) improved acceptability of the diverse components of the programme. Regarding the changes in outcomes associated with participation in the programme, we expected improvements on the five core outcomes comparable in size to those observed in our first trial. In parallel, we aimed to obtain in-depth qualitative information about the new features of the programme, as well as its benefits and challenges, from the point of view of ICDs. In reporting on this quasi-experimental intervention study we follow the Template for Intervention Description and Replication Checklist (TIDieR) guidelines [24]. The data presented here were collected between 2017 and 2020.

#### Sample

A convenience sample of ICDs has been recruited through service providers in the field of dementia (Alzheimer Association, home care nurses, memory clinics, day-care centres) operating in two regions of the French-speaking part of Switzerland (Fribourg and Jura). Participants volunteered for a psycho-educational intervention focusing on stress management, along with pre- and post-intervention interviews. Inclusion criteria were 1) being a regular informal caregiver (at least once a week on average) of a person living with a diagnosis of dementia (as reported by the ICD based on a physician evaluation), 2) caring for this person since at least 6 months. Exclusion criteria were 1) insufficient French-language skills, 2) low caregiver burden (score below 10 on the Zarit Burden Interview), and 3) no memory or behavioural problems in the PwD. Participants were requested to pay a course fee of 210 Swiss Francs (230 US$), which could be waived upon request (this occurred for two ICDs).

#### Procedure

**Fig. 2:**
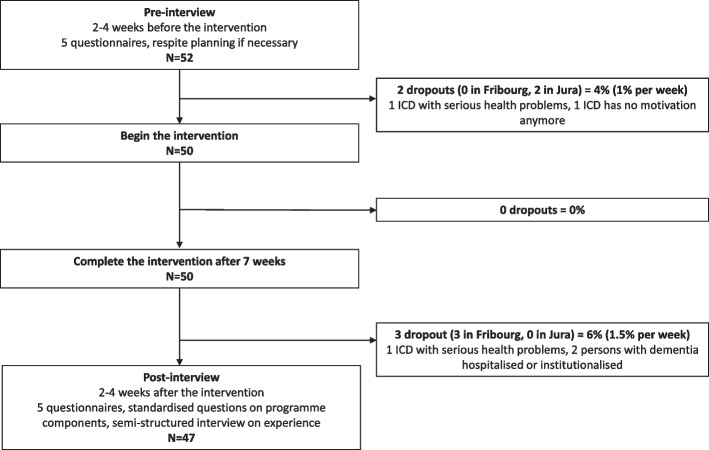
Flow chart of the inclusion in the intervention as well as pre- and post-interview

The study was performed in accordance with the Helsinki Declaration and was approved by the local ethics review board (ISRCTN13512408). Written informed consent was obtained from each ICD following oral and written information provided by a member of the research team. Figure 2 offers an overview of the study procedure.

Before the intervention, during an individual interview with an experienced researcher, participants completed five questionnaires. During the interview, we asked whether participants needed someone else to care for their PwD during the intervention, which we could organise with the local Alzheimer Association, our partner in the project. The ICDs then participated in the intervention described below. After the end of the intervention, they took part in a second interview with the same researcher to complete the five questionnaires again, and report on their experience with the intervention by answering open and closed questions.

#### Intervention

The LFBHB intervention was originally developed by Louise Levesque, Francine Ducharme and their team in the years 2000s, based on Lazarus and Folkman’s transactional theory of stress and coping [25]. The programme aimed at improving the ability of ICDs to cope with the stressful demands of caring for a person with dementia living at home [26]. Its content focuses primarily on 1) the appraisal of stressful situations, and 2) the coping strategies, organised around the five themes presented earlier. Regarding appraisal, participants learn to break down a global situation into specific ones, identify precisely what is stressful, and distinguish between situations or aspects which can be modified and those which cannot. Regarding coping strategies, participants are trained to choose an appropriate strategy depending on whether the situation can be modified or not, to use problem solving techniques for modifiable situations (seven-step procedure), to use reframing for unmodifiable situations (looking at things from another angle to reduce painful emotions), and to seek social support (identify precise support needs and the best persons to address each of them, learn through role playing how to ask efficiently for the needed support). In addition, information is provided on how dementia may affect the communication and relational behaviour of the affected person, and how ICDs may improve their communication skills and prevent tensions, including role-playing exercises about communication. The programme uses a combination of 1) information provision, 2) group discussions, 3) work on personal stressful situations, and 4) exercises at home. The content and methods used in programme are described in detail in Lévesque et al*.* [26]. Participants receive a booklet containing key information and exercise sheets, and the course leaders deliver the intervention according to a detailed manual. A logbook was given to participants to collect notes about difficult and enriching situations in their daily caregiving during two weeks before the start of the intervention. The shortened intervention consisted of 7 weekly group sessions of 3 h each (with a 20-min break in the middle). It was held in a quiet room with easy road and public transportation access, located at the School of Health in Fribourg or at a local day-care centre in Jura between 2017 and 2020. Two nurses and three psychologists with expertise in dementia and work experience with informal caregivers delivered the sessions in pairs for large groups (7–10 participants) or alone for smaller groups. All course leaders had completed a 4-day training related to the intervention programme, had experience in leading the courses and had received supervision from a trained psychotherapist with extensive experience in psycho-educational group interventions. Adherence to the course manual was not assessed as it had already been confirmed in a previous phase [13].

#### Measures

We collected 1) *quantitative measures* for five outcomes, using four questionnaires (one is assessing two outcomes), and 2) *quantitative and qualitative post-intervention reports* on the experience about the programme.

### Questionnaire measures

#### Caregiver’s subjective burden

We measured burden using the Zarit Burden Interview [27], a well-validated and widely used 22-item questionnaire. Responses are provided on a scale from 0 (*never*) to 4 (*very often*). Scores above 18 indicate a heavy burden and scores above 32 a severe burden [28]. The French version has satisfactory psychometric properties: internal consistency is good with α = 0.92, the factor structure is confirmed, and the convergent validity is well established through substantial correlations with the severity of dementia, the level of dependency, the cognitive impairment and the behavioral problems in the PwD, as well as caregiver depression [28].

#### Memory and behavioural problems (MBP) and caregiver’s MBP-related distress

We measured these two outcomes with the Revised MBP Checklist [29], a questionnaire which measures the frequency of 24 MBP in the previous week from 0 (*never*) to 4 (*daily*), and the extent to which this disturbed or upset the ICD between 0 (*not at all*) and 4 (*extremely*). This questionnaire is in French and has satisfactory psychometric properties: internal consistency is good with *α* = 0.84 for PwD behaviour and *α* = 0.90 for ICD reaction, the factor structure is confirmed, and the convergent validity is well established through correlations with caregiver depression and burden, as well as PwD cognitive impairment [29].

#### Caregiver’s psychological distress

We used the short version of the Ilfeld Psychiatric Symptoms Index [30], a questionnaire asking participants to rate 14 symptoms related to depression, anxiety, anger and cognitive disturbance, on a 4-point scale from 1 (*never*) to 4 (*very often*). The psychometric properties are satisfactory for the English [30] and French [31] versions, with good internal consistency (*α* = 0.91), subscale structure confirmed by factor analysis, and convergent validity demonstrated through associations with seeking professional help or hospitalization for emotional problems, as well as use of psychoactive drugs [30].

#### Caregiver’s self-efficacy

We measured self-efficacy as initially suggested by Bandura [32], with a visual analogue scale ranging from 0 (*no confidence at all in my ability to assume my caregiver role*) to 10 (*full confidence*).

### Post-intervention reporting on the experience of the programme

#### Quantitative

We assessed the perceived usefulness of the four strategies (communication, modifying unhelpful thoughts, problem solving and support seeking) and seven methods (short video films, information provided by the course leaders, course booklet, working on personal situations, large group exchanges, small group exchanges and exercises at home) used in the programme. For each, there were three items: 1) I found it interesting, 2) I found it useful, and 3) It helped me in my daily life. Answers were to be given on a 5-point scale: 0 *Not at all or very little*, 1 *A little*, 2 *Moderately*, 3 *Very*, 4 *Extremely*. As they correlated highly with each other (in nearly all cases Spearman’s *ρ* > 0.50) we created a new variable which was the average of the three items for each strategy or method. Regarding the four strategies, we also asked the two following questions: 1) I found it difficult to understand, 2) I found it difficult to apply; With answers on the same 5-point scale.

#### Qualitative

Semi-structured interviews were performed with participants within two weeks after the end of the programme. Interviews were performed as soon as possible after the intervention to capture caregivers’ experiences while minimizing retrospection effects and interference of other events, in view of the frequent changes typical of dementia caregiving. Interviews took place at the location where the course was given or in participants’ homes and were conducted face-to-face by one of two scientific collaborators with a master’s degree in psychology (MC, ET) not involved in providing the programme. The interview guide focused on benefits and negative aspects of the intervention as well as participants’ experience regarding the course, including adapted or new elements such as the course material (e.g. films) and the organisation of the course. Fieldnotes were taken during the interviews to document information and observations relevant for data analysis. Interviews were audio recorded, verbatim transcribed and anonymized.

### Data analysis

#### Quantitative

We used descriptive statistics to provide general information on the study outcomes (median, Q1 and Q3, as 2 out of our 10 variables significantly departed from normality, namely self-efficacy at pre-test and psychological distress at post-test) and to analyse the quantitative post-intervention reports (median, Q1, Q3 and boxplots as they are measured on ordinal scales). For the five quantitative outcomes, changes between pre- and post-intervention were tested with Wilcoxon tests conducted on each outcome, with α set at 0.01 after Bonferroni correction. To allow comparisons with previous studies, the effect size (Cohen’s d) was computed. We also tested differences between the original programme [15 sessions, [13]] and the shortened one regarding changes between pre- and post-intervention with Mann–Whitney tests conducted on the five change scores, again with α set at 0.01.

#### Qualitative

The coding system developed during the first pilot study was used to code the qualitative interviews [13]. This coding system was developed by following the Mayring [23] approach for summarizing content analyses with inductive category assignment. The interviews in the current study were analysed and reviewed to evaluate if all quotes could be assigned to a code and category of the coding system developed earlier or if new codes or categories needed to be added. Additional categories and codes were created with the Mayring [22] inductive summarizing approach to assign in-depth information about the adapted and new elements of the course (the different steps of the approach are described in [13]). Coding was performed by two coders (SK, ET) who regularly discussed the evolving codes and coding system. Regular meetings within the research group were held to discuss findings and discrepancies, in particular during interview coding with the pilot study coding system. Atlas.ti version 9 analytical software was used to facilitate the analysis process.

## Results

### Feasibility

#### Recruitment process

The new recruitment strategy based on our presentation video elicited strong interest from diverse providers of support for ICDs and led to five presentations in diverse organisations (2 in homecare centres, 1 in a memory clinic, 1 in a day-care centre, 1 at the local Alzheimer Association), as well as three presentations for the general public involving about 300 participants in total. The video link (https://tube.switch.ch/videos/f8bfeb3c, with limited visibility) already had more than 1200 views on the 30th of July 2022. According to informal feedback from recruiters, the video was very useful as it helped them better understand the specificity of the programme and helped them present the course to ICDs. Within the recruitment process, nearly all ICDs who registered for the course had already seen the video, and had no hesitation about participating, nor questions about the programme other than organisational matters such as timing, location or price. None of them decided not to participate due to the length of the course. In 2017–2018, we conducted three such courses and were able to recruit 7 to 9 participants for each of them (in total 23 ICDs), in line with our target (7–10 participants per group). In 2019–2020, due to a reduction in the number of available course leaders and the COVID pandemic, we reduced our target size to 4–7 participants per group while increasing course frequency. During this period, we conducted six courses with 3 to 7 ICDs (in total, 29 ICDs).

#### Dropout rates

Out of the 52 recruited participants, two dropped out just before starting the programme due to serious health problems for the ICD (*N* = 1) or a lack of motivation (*N* = 1). All of the 50 participants who started the programme completed it. Three participants were unable to complete the follow-up questionnaires due to hospitalisation (*N* = 1) or institutionalisation (*N* = 1) of the PwD or unexpected health problems for the ICD (*N* = 1). The remaining 47 participants were included in the final analyses. The dropout rate was thus 9.62%.

#### Participation in the programme sessions

The average participation rate in the programme was very high (95%). All participants took part in at least 5 sessions and 72% of the participants (*N* = 34) did not miss any sessions. The few missed sessions (18) were due to the ICD’s illness, the death of a relative, an unavoidable appointment, professional obligations, the hospitalisation or unexpected severe health problems of the PwD, or planned holidays.

### Sample characteristics

As presented in Table 1, participating ICDs (*N* = 47) were mostly women, and often spouses or children of the PwD, with a median age of 61 years (range 38–83). PwD were more often men with a median age of 78 years (range 55–93). All had a diagnosis of dementia, including 51% Alzheimer’s disease, 34% unspecified or mixed dementia, 9% (*N* = 4) vascular dementia, 4% (*N* = 2) fronto-temporal dementia, and 2% (*N* = 1) Lewy body dementia. The ICDs had been providing care for a median duration of 2 years (range 0.75–10 years), and were currently in charge of the patient for a median of 5 days per week (range 0.5–7 days).

**Table 1:** Descriptive statistics for the study variables at pre-test for the original versus shortened programme

		**Original programme (*N* = 18)**	**Shortened programme (*N* = 47)**	**Tests**	***p*-value**
ICD gender^a^	Female / Male	78% (14) / 22% (4)	83% (39) / 17% (8)	0.23	0.629
PwD gender^a^	Female / Male	39% (7) / 61% (11)	43% (20) / 57% (27)	0.07	0.788
Diagnosis^a^	Alzheimer / Unspecified / Other type	50% (9) / 33% (6) / 17% (3)	51% (24) / 34% (16) / 15% (7)	0.03	0.984
Age ICD age^b^		69.50 (56.50 – 72.25)	61.00 (56.00 – 69.00)	1.51	0.131
Age PwD age^b^		76.00 (70.00 – 81.25)	78.00 (72.00 – 84.00)	1.34	0.179
Duration of illness (years)^b^		3.00 (2.50 – 5.00)	2.00 (2.00 – 4.00)	1.43	0.154
Time per week spent caregiving (days)^b^		6.00 (3.50 – 7.00)	5.00 (1.38 – 7.00)	0.99	0.322
ICD burden (0–88)^b^		42.75 (33.38 – 53.75)	33.00 (23.00 – 42.50)	2.77	0.006
MBP of the PwD (0–4)^b^		1.75 (1.22 – 1.93)	1.42 (1.13 – 1.67)	1.96	0.050
MBP-related distress in ICD (0–4)^b^		1.97 (1.54 – 2.49)	1.92 (1.46 – 2.25)	0.62	0.538
ICD psychological distress (14–56)^b^		27.75 (24.50 – 29.00)	22.50 (19.00 – 28.00)	2.47	0.014
ICD self-efficacy (0–10)^b^		7.50 (5.00 – 8.00)	8.00 (7.00 – 8.50)	2.33	0.020

For all the variables listed, higher scores indicate higher levels. *ICD* Informal Caregiver of a person with Dementia, *PwD* Person with Dementia, *MBP* Memory and Behaviour Problems, *Unspecified* Unspecified dementia, *Other* Other type of dementia, For burden, scores above 18 indicate an important burden and scores above 32 a severe one. ^a^ For nominal variables, descriptive statistics are percentage and (frequency), tests are χ^2^. ^b^ For quantitative variables, descriptive statistics are median (Md), first (Q1) and third (Q3) quartiles, tests are Mann–Whitney (Z)

Compared to participants in the original programme (see Table 1), those in the shortened programme do not differ significantly regarding socio-demographic characteristics (gender and age of the ICD or PwD, type of family bond), nor on variables related to the caregiving role (diagnosis, duration of illness, caregiving time per week).

*Pre-test questionnaire results for the shortened programme.* As shown in Table 1, at pre-test the ICDs’ burden was severe overall, with a moderate frequency of patient MBP and caregiver MBP-related distress. The ICDs reported moderate psychological distress, and a relatively high self-efficacy. The spouse and children ICDs did not differ significantly on any of the five outcomes at pre-test [see Additional file 1].

Compared to participants in the original programme (*N* = 18), those in the shortened programme had significantly more favourable scores on 4 of the 5 outcomes (lower subjective burden, lower frequency of patients’ MBP and caregivers’ MBP-related distress, lower psychological distress and higher self-efficacy). The distributions showed that this difference was mostly due to having a higher proportion of participants with lighter difficulties in the shortened programme, and a lower proportion with severe difficulties. However, the absolute frequencies of participants with severe difficulties were also high in the shortened programme. For example, there were respectively 0 and 11% of participants with moderate burden, 22 and 38% with heavy burden, and 78 and 51% with severe burden, corresponding in absolute frequency to 14 and 24 ICDs with severe burden.

### Changes between pre- and post-intervention scores

Comparing pre- and post-intervention scores for the shortened programme (see Table 2) with Wilcoxon tests, we observed that subjective burden, psychological distress and caregivers’ MBP-related distress decreased significantly, with small to medium effect sizes. There was no significant change in the frequency of patients’ MBP or self-efficacy.

**Table 2:** Median (Md), first (Q1) and third (Q3) quartiles for the study variables, pre-test and post-test

	Pre-test dropouts (*N* = 5) **Md (Q1 – Q3)**	Pre-test completers (*N* = 47) **Md (Q1 – Q3)**	Post-test (*N* = 47) **Md (Q1 – Q3)**	Wilcoxon Z (df = 46)	p-value	Effect size (d)
Burden (0–88)	33.00 (10.50 – 39.25)	33.00 (23.00 – 42.50)	28.50 (22.00 – 36.00)	2.70	0.007	0.32
MBP (0–4)	1.71 (1.09 – 2.04)	1.42 (1.13 – 1.70)	1.29 (1.05 – 1.67)	0.89	0.371	0.14
MBP-related distress (0–4)	1.07 (0.48 – 1.84)	1.85 (1.44 – 2.23)	1.44 (1.03 – 1.94)	2.72	0.007	0.57
Psychological distress (14–56)	23.00 (18.50 – 26.25)	22.50 (19.00 – 28.00)	20.50 (18.00 – 24.50)	3.36	0.001	0.48
Self-efficacy (0–10)	10.00 (9.00 – 10.00)	8.00 (7.00 – 8.50)	8.00 (7.00 – 9.00)	-0.13	0.897	-0.02

For all the variables listed, higher scores indicate higher levels, *MBP* Memory and behavioural problems, for burden, scores above 18 indicate an important burden and scores above 32 a severe burden; among the 5 dropouts, 2 declined to participate before the start of the intervention and 3 failed to provide post-test measures.

Comparing the original and shortened programs on the change scores (differences between pre- and post-intervention scores) using a Wilcoxon test, we found no significant differences as shown in Table 3.

**Table 3:** Median, first (Q1) and third (Q3) quartiles for change scores for original versus shortened programme

	Original programme (*N* = 18) **Md (Q1 – Q3)**	Shortened programme (*N* = 47) **Md (Q1 – Q3)**	Mann–Whitney (Z)	p-value
Burden (0–88)	-4.00 (-11.00 – 2.00)	-4.50 (-9.50 – 2.50)	0.26	0.797
MBP (0–4)	-0.08 (-0.25 – 0.18)	-0.04 (-0.26 – 0.19)	0.07	0.942
MBP-related distress (0–4)	0.01 (-0.28 – 0.30)	-0.40 (-0.94 – 0.38)	1.61	0.108
Psychol. distress (14–56)	-2.00 (-5.25 – 2.25)	-3.00 (-5.00 – 1.00)	0.29	0.775
Self-efficacy (0–10)	0.75 (-0.13 – 1.25)	0.00 (-1.00 – 1.00)	1.72	0.086

For all the variables listed, higher scores indicate higher levels, *MBP* Memory and Behaviour Problems, For burden, scores above 18 indicate an important burden and scores above 32 a severe one.

### Satisfaction with didactic methods and the contents of the programme

#### Quantitative results

**Fig. 3:**
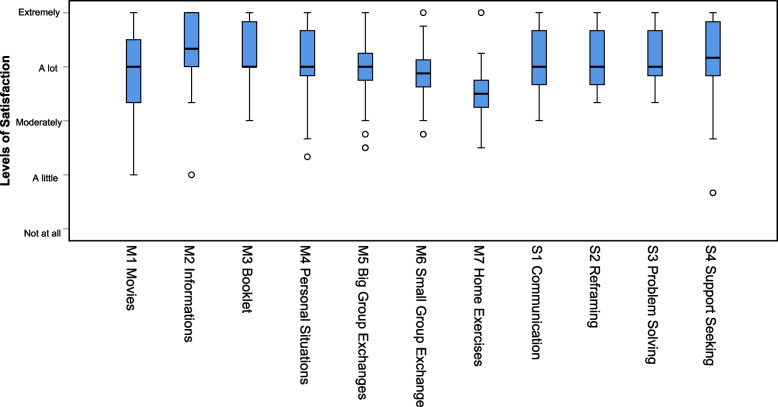
Boxplots for satisfaction with methods (M) and strategies (S), *N* = 47

**Fig. 4:**
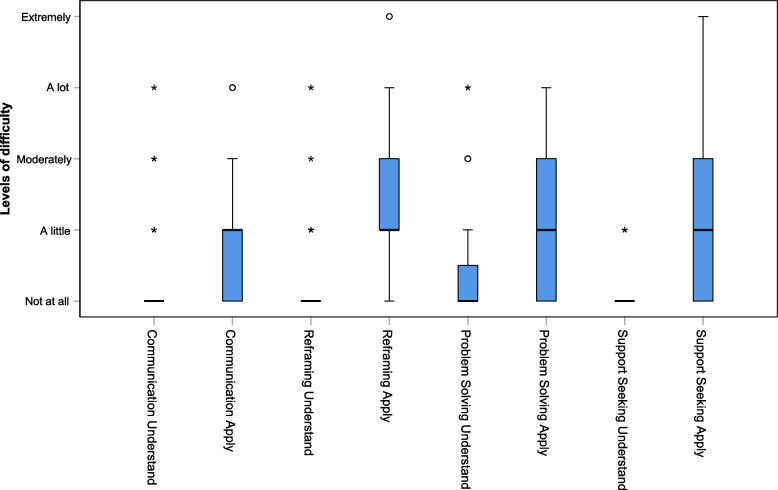
Boxplots for perceived difficulty in understanding or applying strategies, *N* = 47

As presented in Fig. 3, the median value for the seven didactic methods used (films, information provided by course leaders, course booklet, working on personal situations, group exchanges (small / big group), and exercises at home) was close to 3 out of 4, corresponding to the answer “very relevant / useful / helpful in daily life”, with very few answers below 2 (“moderately”). The same was observed for the four types of strategies (communication, modifying unhelpful thoughts, problem solving and support seeking). Figure 4 presents the results for the two questions exploring whether the participants perceived the four contents as difficult to understand or to apply. For difficulties in understanding, all medians were at 0 (“Not at all”) with less than 17% of responses above 0. For difficulties in applying, all medians were at 1 (“A little”) with few responses above 2.

As presented in Table 4, two of the four didactic methods used in the programme elicited more satisfaction in the shortened programme than in the original one. The satisfaction scores for the information provided by the course leaders and the exercises at home were higher in the shortened programme than in the original one, while both groups were similarly satisfied with group exchanges and working on personal situations. Regarding the four strategies taught in the programme (communication, modifying unhelpful thoughts, problem solving and support seeking), satisfaction was significantly higher for problem solving among participants in the shortened programme compared to the original intervention, with no significant differences regarding the three other strategies.

**Table 4:** Median, first (Q1) and third (Q3) quartiles for participant satisfaction regarding original and shortened programs

	Original programme		Shortened programme		Mann–Whitney (Z)	p-value
	Md	Q1 – Q3	Md	Q1 – Q3		
Information (*N* = 18, *N* = 47)	3.00	2.67 – 3.00	3.33	3.00 – 4.00	2.96	0.003
Personal Situations (*N* = 18, *N* = 47)	3.00	2.67 – 3.33	3.00	3.00 – 3.67	1.83	0.067
Group exchanges (*N* = 18, *N* = 47)	3.08	2.67 – 3.75	3.00	2.67 – 3.67	0.37	0.710
Home Exercises (*N *= 18, *N* = 47)	2.75	2.33 – 3.00	3.00	3.00 – 3.67	3.02	0.002
Communication (*N* = 18, *N* = 45)	3.00	2.88 – 3.42	3.00	2.67 – 3.67	0.24	0.808
Reframing (*N* = 18, *N* = 46)	3.00	2.58 – 3.33	3.00	2.67 – 3.67	1.22	0.221
Problem solving (*N* = 17, *N* = 45)	3.00	2.33 – 3.00	3.00	3.00 – 3.67	2.70	0.007
Support seeking (*N* = 18, *N* = 45)	3.00	2.67 – 3.67	3.00	2.83 – 3.67	0.76	0.447

For all the variables listed, higher scores indicate higher levels; Ns are provided for the original (first) and shortened (second) programs; Satisfaction is measured on a scale ranging from 0 = not at all to 5 = extremely, regarding the four didactic methods used (proving information, working on personal situations, group exchanges and home exercises) and the four strategies taught (communication in the context of dementia, reframing, problem solving and support seeking) in the programme

As shown in Table 5, we observed no significant difference between the original and shortened programs regarding the participants’ perceived difficulty in understanding or applying the learned strategies, except for reframing, which was found easier to apply in the shortened programme.

**Table 5:** Median, first (Q1) and third (Q3) quartiles for participants’ difficulty in original and shortened programs

	Original programme		Shortened programme		Mann–Whitney (Z)	*p*-value
	Md	Q1 – Q3	Md	Q1 – Q3		
Understand Communication (*N* = 18, *N* = 45)	0.00	0.00 – 0.25	0.00	0.00 – 0.00	0.62	0.534
Apply Communication (*N* = 18, *N* = 45)	1.00	0.00 – 2.13	1.00	0.00 – 1.00	1.82	0.069
Understand Reframing (*N* = 18, *N* = 46)	0.00	0.00 – 1.00	0.00	0.00 – 0.25	0.46	0.643
Apply Reframing (*N* = 18, *N* = 46)	2.00	1.75 – 3.00	1.00	0.75 – 2.00	2.76	0.006
Understand Problem Solving (*N* = 17, *N* = 45)	0.00	0.00 – 1.00	0.00	0.00 – 0.50	0.87	0.386
Apply Problem Solving (*N* = 17, *N* = 45)	2.00	1.00 – 2.00	1.00	0.00 – 2.00	1.03	0.305
Understand Support seeking (*N* = 18, *N* = 45)	0.00	0.00 – 0.00	0.00	0.00 – 0.00	1.61	0.108
Apply Support seeking (*N* = 18, *N* = 45)	1.00	0.75 – 2.00	1.00	0.00 – 2.00	1.06	0.288

For all the variables listed, higher scores indicate higher levels; Ns are provided for the original (first) and shortened (second) programs

#### Qualitative results

The 47 participants who were included in the quantitative analysis also took part in interviews lasting on average 35 min (range 11–71 min). All quotes regarding the benefits of the programme could be allocated to one of three categories developed during the first qualitative evaluation of the programme: “1) *Sharing experiences and strategies,* which is about participants learning from each other, comparing their situations with others and becoming strengthened in their way of handling the situation; 2) *Being in the same boat*, namely feeling understood, less alone and connected to the other ICDs because they are in similar situations; 3) *Being able to cope*, as the programme empowered participants to develop strategies, which helped them manage their challenging situations” [13], p. 7]. The results of the current qualitative analysis on the shortened programme are consistent with those of the original analysis, which are described in more detail in Pihet et al. [13].

To obtain in-depth information about new and adapted elements (e.g. the videos), we asked additional questions about the course material and organisation. Linked to these questions, three new categories emerged: 4) *Having something to rely on* contains results regarding the importance of having approaches or materials which are supportive in daily life, 5) *Learning through multiple perspectives and channels* describes how participants learn through different didactive methods and perspectives, and 6) *Organising the intervention* provides information on the frequency and length of the sessions as well as important aspects regarding group leaders. In the upcoming section, we present the results for these three new categories.

**Having something to rely on** describes the importance of having a structured and practical method or material which is useful for participants in their daily lives, in order to solve their problems and/or to inform other persons involved in caregiving. Even though participants needed time and practice to become familiar with the material and its content, they were then able to use it by themselves to find solutions when facing a difficult situation with their sick loved one.

*“(…) The method was very useful... I found it was very structured and we systematically came back to it! The facilitators were really very clear on this and it was really a lifebuoy! It helped us not to drown in all the challenges, there was really something we could rely on.”*

*“I read passages [of the course booklet] again. I say to myself "OK, how can I try to solve this problem?" and then I try to apply the method one step after the other, because I know I have the tendency to jump to a solution without taking my emotions into account.”*

In addition, participants perceived the course booklet and videos as helpful during the course as well as afterwards, as a resource to come back to. They described as reassuring the fact of knowing that they have all the material available in case they misunderstood or forgot any information given during the course, or in case they need to solve a new problem. They appreciated being able to read the course booklet or watch the videos directly after the course sessions, sometimes several times, to deepen their understanding of the content and recall the different steps of the method. Most participants reported reading the booklet again after each session, while some planned to read it again later when their relative's illness would have progressed.

*“There are many things in the course, so having something on paper that you can refer to allows you to go back to it, to delve a bit further in it, to say: "Oh, I didn't understand it in that way, maybe I should have understood it differently". While we're talking about all this, sometimes we may miss a little step or a little part, so that we don't always understand perfectly, and then by reading it again we can complete these notions.”*

Participants also used the course material to share the information and their new knowledge with other family members involved in the caring situation.

*“My father [who also participated in the course] gave his brochure to my sister, who read it. Even if she only had the theory without the practical input, this allowed her to make it her own.”*

A logbook was given to participants to collect notes about difficult or enriching situations in their daily caregiving during the two weeks preceding the course. It was often described as useful by the participants, as it helped them to reflect on their situations and to remember it during the course.

*“(…) For analysing concrete cases, it was very useful. So, instead of improvising, I was able to use quotes that I had written down in the notebook.”*

For each difficult situation, participants were asked to rate the stress they experienced in that situation. At first, some found it difficult to assess the intensity of their stress, as they were not used to this kind of assessment, but they later became familiar with this evaluation.

*“The first time I saw it I thought: “How do I fill this in?” Well, it wasn't easy for me to say how I felt either. This scale with values is tricky at the beginning, if you're not used to identifying what can be painful or not. If you haven't thought it through before, it's a bit tricky to fill out.”*

**Learning through multiple perspectives and channels** was also appreciated by the participants. They appreciated the diverse intervention material such as the course booklet and the videos, as well as the didactic methods, which helped them better understand the content of the programme. The course booklet was valued for its diversity of theoretical information, guided exercises, stories of former participants and in-depth information on certain themes. The content of the booklet was generally described as broad in content and easily understandable, with clear information given in simple terms.

*“That's good! It's great, I always have it with me. I enjoyed reading either the stories or the explanations, and then I know I can come back to them! To the guided exercises to analyse the situation or to ask for help, for example.”*

Participants enjoyed having both written and audio presentations of the theory as well as being able to read or listen to stories of former participants. The latter were described as helpful in all didactic methods used throughout the course, namely in the booklet, videos, group discussions and practical exercises.

*“(…) There are several course materials, which I found very good. We can also share our experience, [then] there were the videos and then there was the booklet, I found… it's just great! It's pure happiness, in any case, to be able to follow a course like that.”*

Stories of former participants shown in the videos were often described as interesting and authentic (i.e. referring to real-life situations) and a help for understanding the approach taught in the programme. For groups with fewer participants, the many stories provided in the course materials were particularly valued as additional examples of other caregiving situations.

*“To know more about memory and its disorders, I found it very interesting even if I already had some knowledge about it, but [it was good] to see it again… and then I find this video really great, it really helps! I found it [the stories of former participants] moving, (…) I liked the positive attitude of these people, as they spoke about their situation and described it in their own very clear words, inspiring words. Yes, I liked it, I thought it was very good!”*

Regarding group discussions, ICDs found it helpful to listen to the many different experiences and to see how various ideas from participants or group leaders contributed to possible solutions. Participants also appreciated having the opportunity to choose, out of many different situations, the one to be analysed and handled in this session.

*“(…) Here [in the large group], we had all the situations. I found it was good to know what difficulties the others might be experiencing. And we always wanted to find solutions to help them. We feel like helping others!”*

Working in smaller groups was also valued, particularly after having had some time to establish trustful relationships. It was sometimes considered easier to address sensitive topics or to work towards solutions than in a larger group.

*“(…) [During group discussions] I sometimes had difficulty expressing myself, and then if there were two or three of us it was much easier to express myself.”*

Participants described role-playing as useful to exercise new ways of doing things. It allowed them to better understand and integrate the theory. Some however did not dare engage in this activity, as they found it stressful to perform in front of the group.

*“She really played the game of speaking with a person suffering from Alzheimer’s for a few minutes, without saying anything [meaningful] but in a very positive mood, and this was completely fine. It helped me. Now, sometimes when my husband wants to tell me something, it is almost a game, I play along with him. Before it was more annoying when he didn’t succeed in asking me something. Now, sometimes it can almost become a game thanks to the role play I saw.”*

*“I didn't participate, I thought "I hope it's not my turn" (laughs) as I generally don't like it. But the situation I observed gave a result. Putting people in the situation, now I understand what role-playing is for (laughs).”*

**Course organisation - Length of the sessions.** Most of the participants appreciated the length of the sessions (three hours), as it allowed them to find their way into the subject and to move forward in the topics. However, some participants also found it was rather time-consuming, particularly for employed ones.

*“I found it [three hours] was great. Maybe three hours is a bit long. It's fine if you don't work, but if you work, that means three hours every week, which is quite a lot. It takes a whole afternoon, in the end.”*

Participants reported that the three hours passed quickly but that they would not want longer sessions, as the emotional load and the intensive involvement during the course take a lot of energy. The 20-min session break was important, to have time to rest, but also to have more informal discussions and to get in contact with each other.

*“Because it's really intense! Emotionally too, for everyone. It stirs things up, so I think that the sessions should not be too long either. It was OK like that.”*

**Course organisation - Number of sessions and frequency.** The majority of participants were comfortable with the six weekly sessions and the follow-up session four weeks after session six. Having one week between each session and in particular four weeks between session six and seven allowed them to exercise the systematic method in their daily life.

*“Then I had plenty of time to meditate, from one week to the next! I was thinking about it. Maybe others don't do it but for me it was important to do it to really absorb it.”*

Most participants said they would like regular booster sessions after the end of the programme. Having created trusting bonds during the course, they wanted to see each other again and hear news from the other participants, as well as to have further opportunities to discuss difficult situations and benefit from the experiences and opinions of the others.

*“And then, in two or three months, [I know] there's a meeting again, and I think that's very important for me. Where you can come with a little thing, a little problem… what takes the most out of you. I think that's important.”*

**Course organisation - Group leaders.** The participants valued the competencies and experience of the group leaders in supporting ICDs. They felt that group leaders listened to them and understood them, as well as provided them useful advice and information that was easy to understand. Many participants enjoyed that group leaders gave guidance with a clear structure to analyse and handle difficult situations while being flexible in adapting the method according to the participants’ needs. They also noticed the importance of giving everyone time to speak, which group leaders took care of. When two group leaders were present, participants enjoyed the complementarity between them.

*“And their [the course leaders’] individualised attention to each person, and they have the capacity to bring out our own resources. There are no recipes and we can go at our own rhythm. Yes, that impressed me a lot...”*

*“They put themselves at our level and adapt to our feelings too, we could really see that there was a lot of listening and then that they understood what was behind it too, that was beautiful. And they were very flexible in their way of functioning because sometimes they had planned a programme and then, because of what we were saying, what we were bringing, they had to change their programme and really they were flexible in doing that.”*

## Discussion

This study illustrates the relevance and also the complexity of a development process following the MRC framework’s new recommendations, which put a stronger focus on “understanding how and under what circumstances interventions bring about change” [20], p.2]. Our development of a psycho-educational group intervention designed for informal caregivers of persons with dementia first identified barriers to participation, namely the length of the programme and that recruiters needed self-explanatory material to present the programme to ICDs [see also [21]]. In a second step and in line with MRC recommendations of a “strong and early engagement with patients, practitioners, and policy makers” in order to “deliver solutions for real world practice” [20], p.2], we involved diverse stakeholders in the process of shortening the intervention and developing a recruitment video: five ICDs, four leaders of the LFBHB programme and seven multidisciplinary experts in the field of dementia caregiving. This participatory phase led to a consensus that stories of former participants were essential in facilitating transfer of the course content to ICDs’ daily life (in order to maintain the effects despite shorter duration) and in presenting the programme to possible participants. Indeed, participants reported that they highly valued these stories which provided inspiration and encouragement. Participants also said that the programme corresponded to what they expected based on the recruitment video. After having seen this video, ICDs had very few questions about the programme or doubts about its relevance for them, while they often had such concerns when we didn’t use it. In line with Bandura’s social learning theory [33], using stories of former participants explaining how they used the relevant coping strategies taught in the course and which benefits they gained from it, was very helpful in engaging ICDs in the course and in strategy use. This process was complemented by the regular group exchanges focused on participants’ experiences while using the strategies in their daily life, which also provided important social learning occasions as they elicited strong attention, provided models that are easy to identify with, and included many stories of how participants were reinforced after using a relevant strategy.

One key result of this study is that shortening the programme to 21 h instead of 30 and to seven rather than 15 weekly sessions increased accessibility while it did not reduce the benefits associated with participation. With the shortened programme, none of the ICDs which contacted us for more information decided not to participate due to the length of the course, while this happened frequently with the original programme. Although the participants in the shortened programme had more favourable scores than those in the original one on most outcomes before the intervention, and thus less room for improvement, we observed substantial improvements in three out of five outcomes, in line with expectations and with improvements observed with the original programme. The refined intervention even led to a significant increase in participants’ satisfaction regarding information provided by the course leaders, exercises at home and problem solving, as well as less difficulty in applying reframing, an essential component to reduce the stress and psychological distress of ICDs [34]. Dropouts were also reduced compared to the original programme (during the intervention: 0% compared to 21%; including pre- and post-test: 10% compared to 31%), and were similarly related to critical events affecting the ICD or the PwD [13]. These results are in line with the growing evidence that low-intensity psycho-educational interventions can effectively support older adults with mild-to-moderate mental health problems while fostering engagement with treatment [35]. Other psycho-educational group interventions for ICDs focusing on stress management such as “Coping with caregiving” [36], have shown similar, although smaller, effects on depression (*d* = -0.36; in our study *d* = -0.48 for psychological distress) and stress reactions related to the behaviour problems of the PwD (*d* = -0.22; in our study *d* = -0.57). This other programme was slightly longer than ours (24 h instead of 21) and spanned over 13–16 weeks instead of 9–11 weeks, which could explain their somewhat higher dropout rates (15% instead of 10%).

Another interesting finding is that, compared to participants in the original programme, those in the shortened one had more favourable scores on most outcomes before the intervention (burden was 23% lower, psychological distress 15% lower, and self-efficacy 19% higher). Recruiting a broader range of ICDs was possibly facilitated by our use of a new strategy: While the first participants were recruited mainly through health care professionals with the support of flyers, in the current study we regularly published information about the intervention in the local media and referred interested persons to a short video illustrating its content. This video was reported very helpful by the recruiters and the ICDs. Our novel strategy helped recruit participants which were not yet in contact with professional support providers and possibly were less burdened.

### Limitations

The main limitation of this study is the absence of a control group, and further research should include a randomized-controlled trial to test the efficacy of this psycho-educational intervention. A second limitation is the lack of a follow-up evaluation, which could reveal additional benefits, as suggested by informal feedback from the participants who came to booster sessions organised three months after the end of the intervention. Many described a more relaxed and accepting attitude towards the person with dementia and particularly his or her difficult behaviours, fostering positivity and reciprocity in the relationship, and in turn reducing feelings of loss and isolation in the ICD. A third limitation is that we did not measure stress levels or the participants’ use of coping strategies, outcomes which would have provided relevant information about the mechanisms of change at play. We decided not to do this, because highlighting the ongoing processes over the course of the intervention would require measuring these variables regularly in everyday life, as was done in our pilot study of the original programme [13]. This procedure was however experienced as tedious for many ICDs. As we did not want to overburden them, we further explored the change processes using qualitative interviews conducted before, during and after the intervention. The analysis of this material is currently underway. A fourth limitation is our modest sample size which precludes conducting subgroup analyses to identify which ICDs may benefit the most from such an intervention. Possible factors, such as relationship type (i.e. child versus spouse caregivers) and other factors identified in the ongoing qualitative analysis (e.g., readiness to change) need to be taken into account in the next research steps.

### Strengths

The main strength of this study is the participatory cultural adaptation of the intervention, which led to innovative solutions such as the use of filmed stories of former participants to densify the intervention. The latter as well as the course booklet were highly appreciated by the participants and often used to raise awareness among other members of the family, an unexpected additional advantage of the intervention. A second core strength is the use of mixed methods, showing converging quantitative and qualitative results about the benefits of the intervention for ICDs, and providing insights on possible change mechanisms: The highly structured content of the intervention, supported by the diverse course materials (e.g. booklet, videos) and the various approaches used to learn new strategies as from the participants’ real-life situations, helped achieve the empowerment of the ICDs. The participants were able to develop more efficient ways of coping with the daily stress of caregiving.

## Conclusion

Aiming at the cultural adaptation of the psycho-educational intervention “Learning to feel better… to help better”, we shortened and densified this programme, resulting in increased acceptability and participants’ satisfaction, while maintaining the associated benefits in terms of ICDs’ quality of life. These achievements are highly favourable for further implementation, paving the way for a pragmatic randomized-controlled trial to evaluate the efficacy of this intervention, identify participants’ characteristics associated with larger benefits and deepen our understanding of the intervention’s change mechanisms. Challenges on this road include identifying the best professional profile for the future course leaders and developing a relevant training curriculum, as well as finding an efficient strategy for ensuring treatment reliability on the long run while facilitating implementation.

### Supplementary Information

**Additional file 1. **Median (Md), first (Q1) and third (Q3) quartiles for pre-test questionnaire results for spouse and child caregivers

## Data Availability

The datasets used and/or analysed during the current study are available from the corresponding author on reasonable request.

## Abbreviations

LFBHB: Learning to feel better… to help better
ICD: Informal caregiver(s) of a person with dementia
MBP: Memory and behavioural problems
MRC: Medical research council
PwD: Person with dementia
TIDieR: Template for Intervention Description and Replication Checklist
