# Supplementary material for: Adaptation of a psycho-educational group programme to improve coping in dementia caregiving: a feasibility study with mixed-methods
Source: BMC Geriatr. 2024 Feb 27;24:197. doi: 10.1186/s12877-024-04815-7 (PMC10900645; doi:10.1186/s12877-024-04815-7)
Supplement: Supplementary file 1 — Additional file 1. Median (Md), first (Q1) and third (Q3) quartiles for pre-test questionnaire results for spouse and child caregivers [file 12877_2024_4815_MOESM1_ESM.docx]

**Additional file 1.** Median, first (Q1) and third (Q3) quartiles for pre-test questionnaire results of spouse and child caregivers

|  | Spouse caregivers (N=24)  Md (Q1 – Q3) | Child caregivers (N=21)  Md (Q1 – Q3) | Mann-Whitney (Z) |
| --- | --- | --- | --- |
| Burden (0-88) | 38.75 (26.13 – 46.50) | 31.00 (21.50 – 36.00) | -1.84 |
| MBP (0-4) | 1.37 (1.08 – 1.65) | 1.46 (1.26 – 1.66) | -0.99 |
| MBP-related distress (0-4) | 2.03 (1.78 – 2.25) | 1.71 (1.24 – 2.36) | - 1.13 |
| Psychological distress (14-56) | 23.75 (21.00 – 28.88) | 21.50 (19.00 – 25.50) | -1.45 |
| Self-efficacy (0-10) | 8.00 (7.00 – 9.00) | 8.00 (7.25 – 8.00) | -0.48 |

*Note.* For all the variables listed, higher scores indicate higher levels; MBP: memory and behavioural problems;

for burden, scores above 18 indicate an important burden and scores above 32 a severe burden
